# Supplementary material for: Integrative functional genomics identifies regulatory mechanisms at coronary artery disease loci
Source: Nat Commun. 2016 Jul 8;7:12092. doi: 10.1038/ncomms12092 (PMC4941121; doi:10.1038/ncomms12092)
Supplement: Supplementary Information — Supplementary Figures 1-20 [file ncomms12092-s1.pdf]

Supplementary Fig. 1

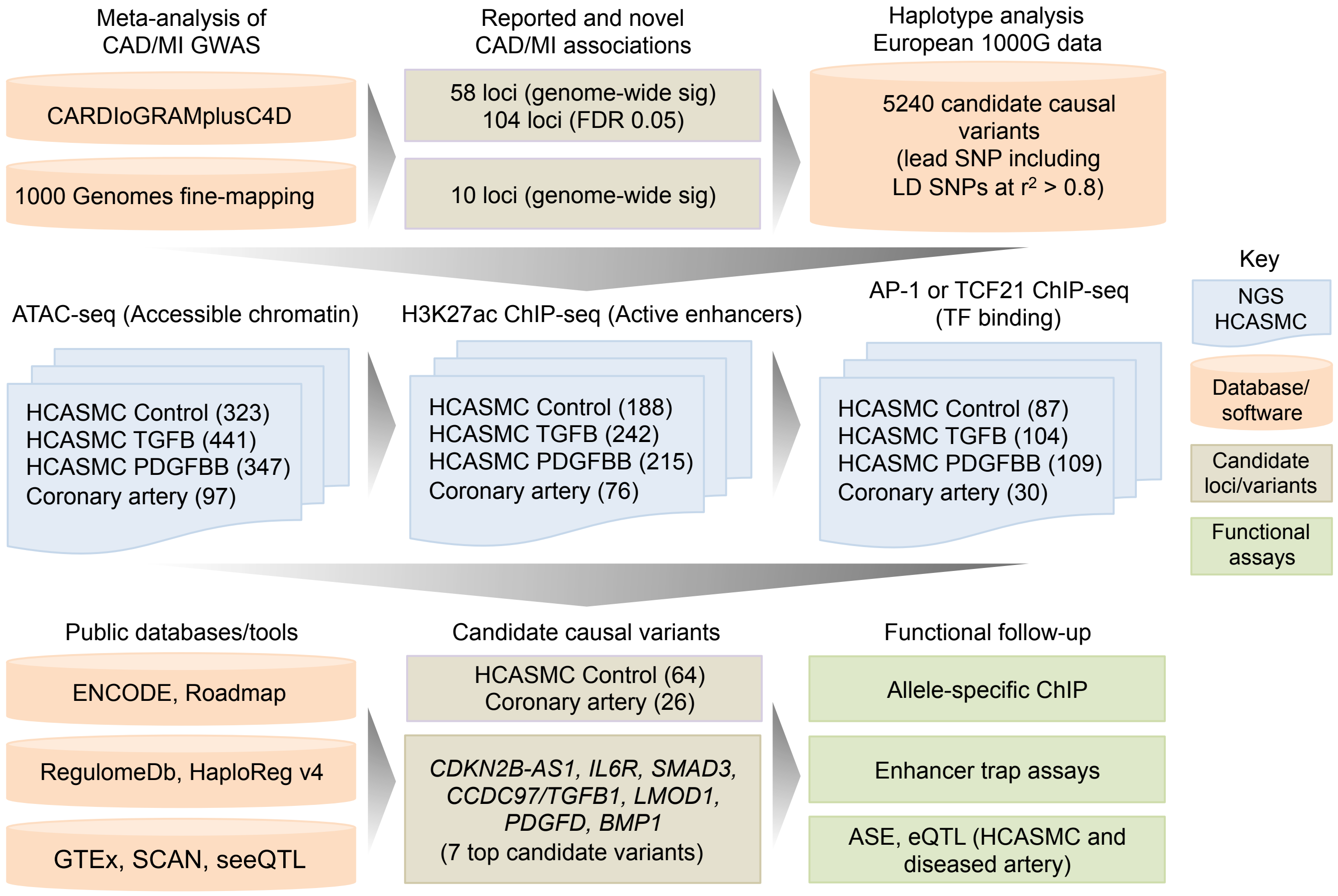

Supplementary Figure 1. Workflow for prioritization of CAD associated regulatory variants. Lead SNPs identified from the CARDIoGRAMplusC4D meta-analysis of GWAS for coronary artery disease, including recent 1000 Genomes fine-mapping results. Proxy SNPs in high linkage disequilibrium (LD) at r2>0.8 were identified using 1000 Genomes phase 1 data in Europeans. These 5240 variants were then mapped to ATAC-seq peaks of accessible chromatin peaks, followed by H3K27ac ChIP-seq peaks of active enhancers, and TCF21 or AP-1 ChIP-seq peaks for transcription factor binding in HCASMC. The resulting variants in the combined overlap (87 using HCASMC control ATAC-seq; 104 using HCASMC + TGFB ATAC-seq; 109 using HCASMC + PDGFBB ATAC-seq; 30 using ex vivo normal coronary artery ATAC-seq) were also further annotated using public databases for regulatory variation, including ENCODE and Roadmap datasets, HaploReg v3 (for motif disruption analysis and protein binding) and RegulomeDb (using priority score cut-off ≤4). Variants were also queried in public eQTL databases including GTEx, SCAN, and seeQTL. Top 7 variants were selected for functional validation using allele-specific ChIP (haploChIP), allele-specific enhancer trap assays using luciferase reporters, and allele-specific expression imbalance assays and eQTL analysis in HCASMC and diseased arterial tissues (STARNET).

Supplementary Fig. 2

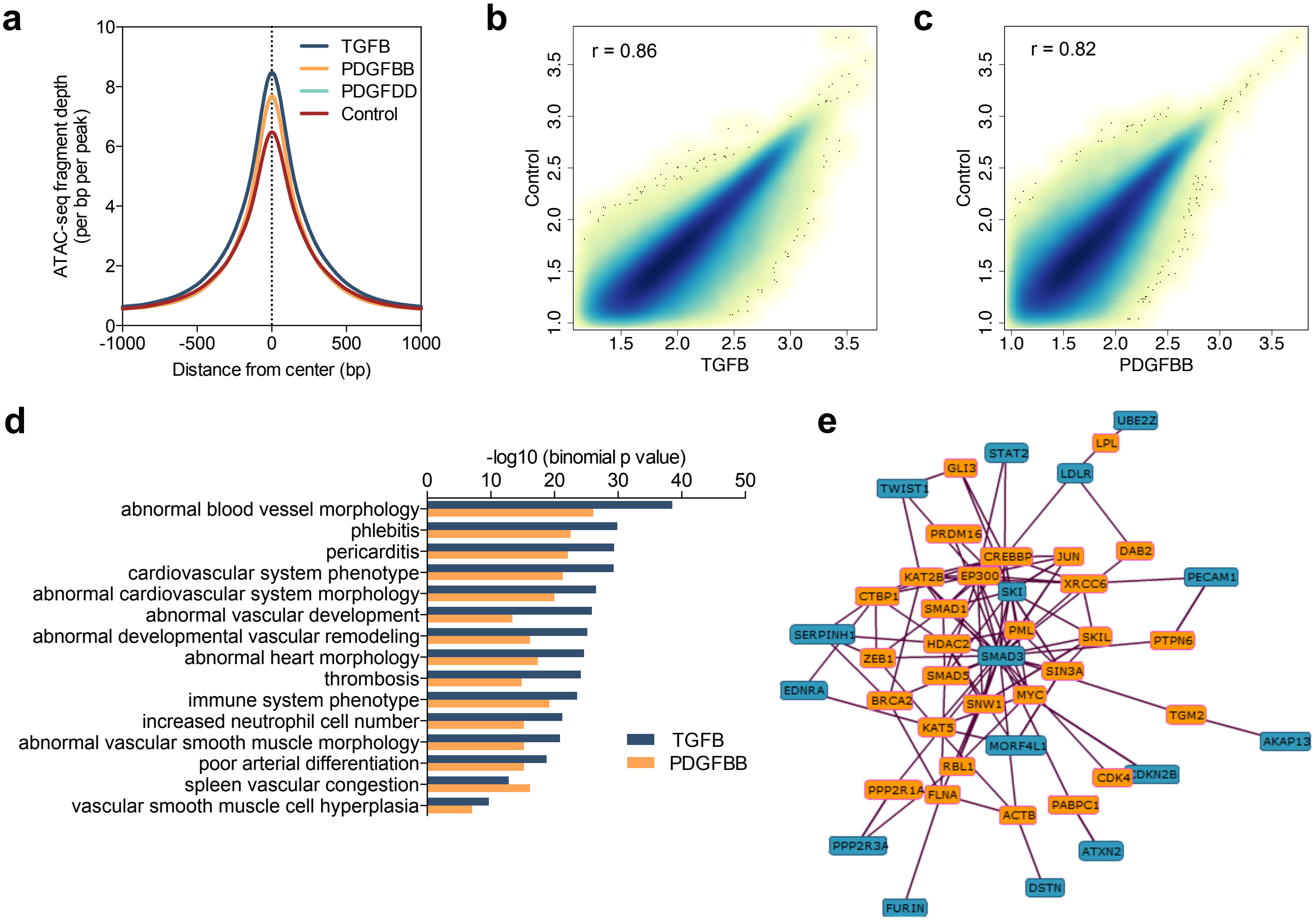

Supplementary Figure 2. Annotation of stimulated HCASMC ATAC-seq peaks. (**a**) Mapped ATAC-seq fragments were normalized and tag densities determined for each condition, plotted within a 1000bp window relative to the center of open chromatin peaks. Values represent n=2 biological replicates per condition. (**b**) Scatter plot and Pearson correlation of ATAC-seq tag densities in representative serum-free (Control) versus TGF-β1 stimulated conditions. (**c**) Scatter plot and Pearson correlation of ATAC-seq tag densities in representative serum-free (Control) versus PDGF-BB stimulated conditions. (**d**) Results of mouse phenotype enrichment analysis (using GREAT) of HCASMC open chromatin peaks stimulated with either TGF-β1 or PDGF-BB and overlapping the entire GWAS catalog. P-values represent Bonferonni corrected binomial p-values for each condition using the whole genome as a background. Similar results were observed from n=2 biological replicates per condition. (**e**) Functional association network (FAN) for open chromatin regions overlapping CAD loci. Blue boxes represent seed genes in queried regions and orange boxes represent significantly connected genes, all of which were identified to interact from 6 protein-protein interaction (PPI) databases, using a z-score significance threshold > 2.25. SMAD3 was identified as a highly connected CAD gene. Also, the AP-1 gene JUN was connected to a number of other CAD associated genes, including SMAD3 and the TGF-β transcriptional co-repressor, SKI. Similar results observed from n=10 biological replicates in HCASMC cultured under normal growth conditions.

Supplementary Fig. 3

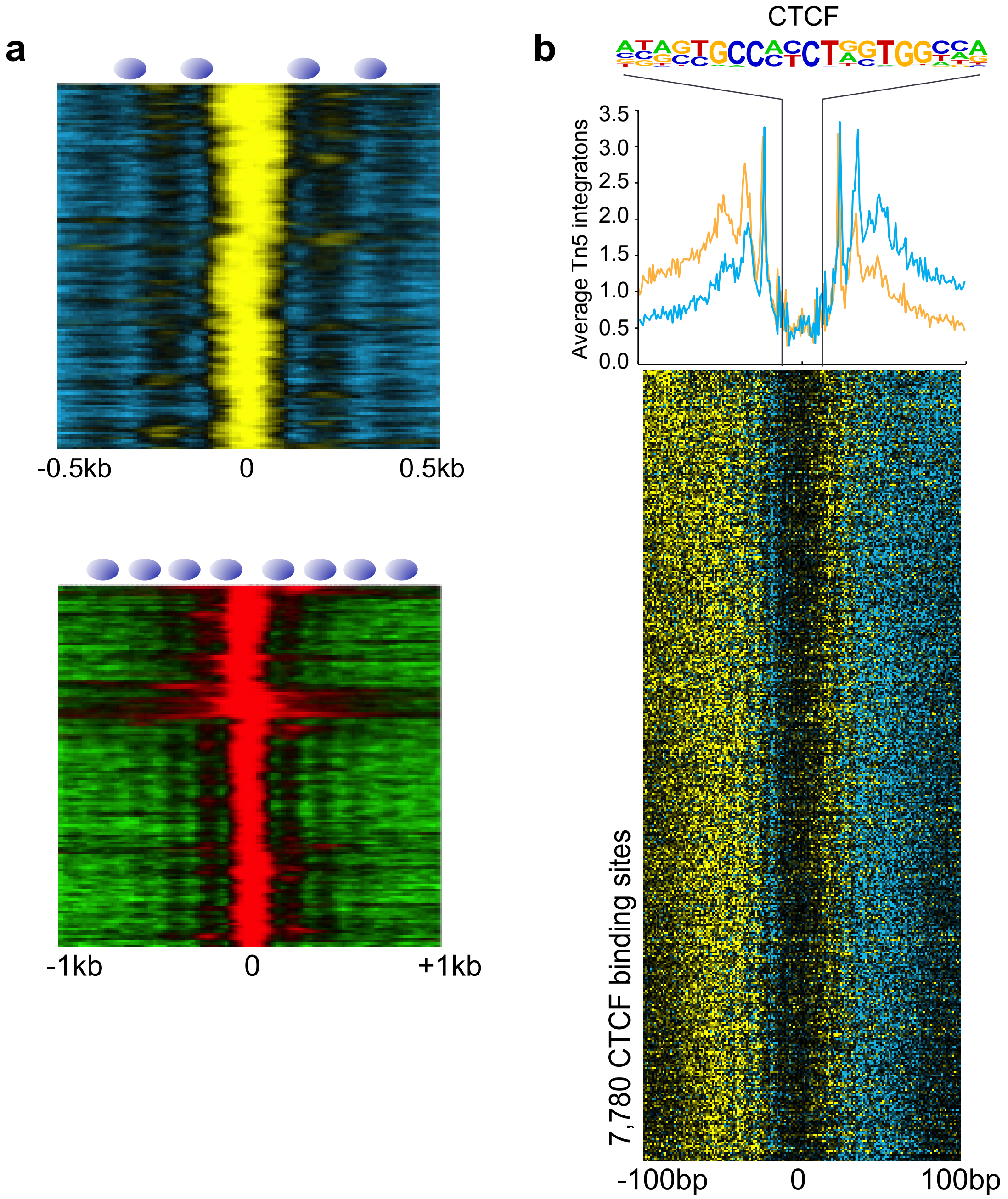

Supplementary Figure 3. Footprinting of HCASMC open chromatin regions. (**a**) Heatmap of HCASMC ATAC-seq open chromatin peaks centered on the consensus CTCF binding site (from JAS-PAR database) showing the organization and positioning of multiple nucleosomes (blue ovals) within a 0.5kb window (top panel) or 1.0kb window (bottom panel). (**b**) Histogram and heatmap of Wellington footprinted HCASMC ATAC-seq open chromatin regions centered on consensus CTCF binding site (from ChIP-seq experiments), showing increased Tn5 transposase integrations outside the CTCF motif, as represented by positive strand (yellow) and negative strand (blue) imbalance. (**a-b**) Similar results observed from n=10 biological replicates in HCASMC cultured under normal conditions.

Supplementary Fig. 4

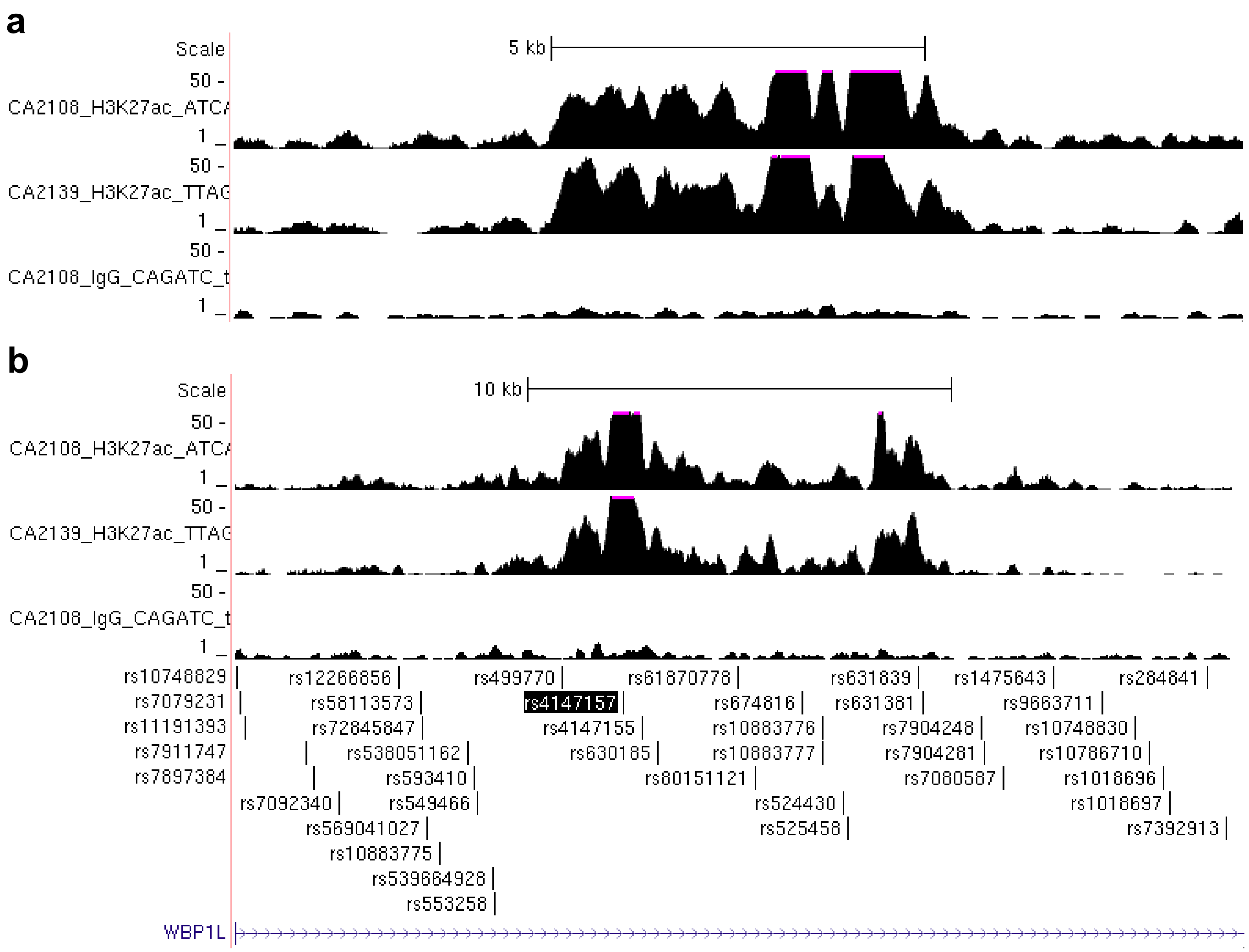

Supplementary Figure 4. Top scoring HCASMC H3K27ac marked super-enhancers. (**a**) Super enhancer encompassing ~5kb at chr6:30,741,369-30,754,681 upstream of the FLOT1 gene. (**b**) SNP rs4147157 (marked black) overlapping HCASMC H3K27ac marked super-enhancer at the WBP1L locus.

Supplementary Fig. 5

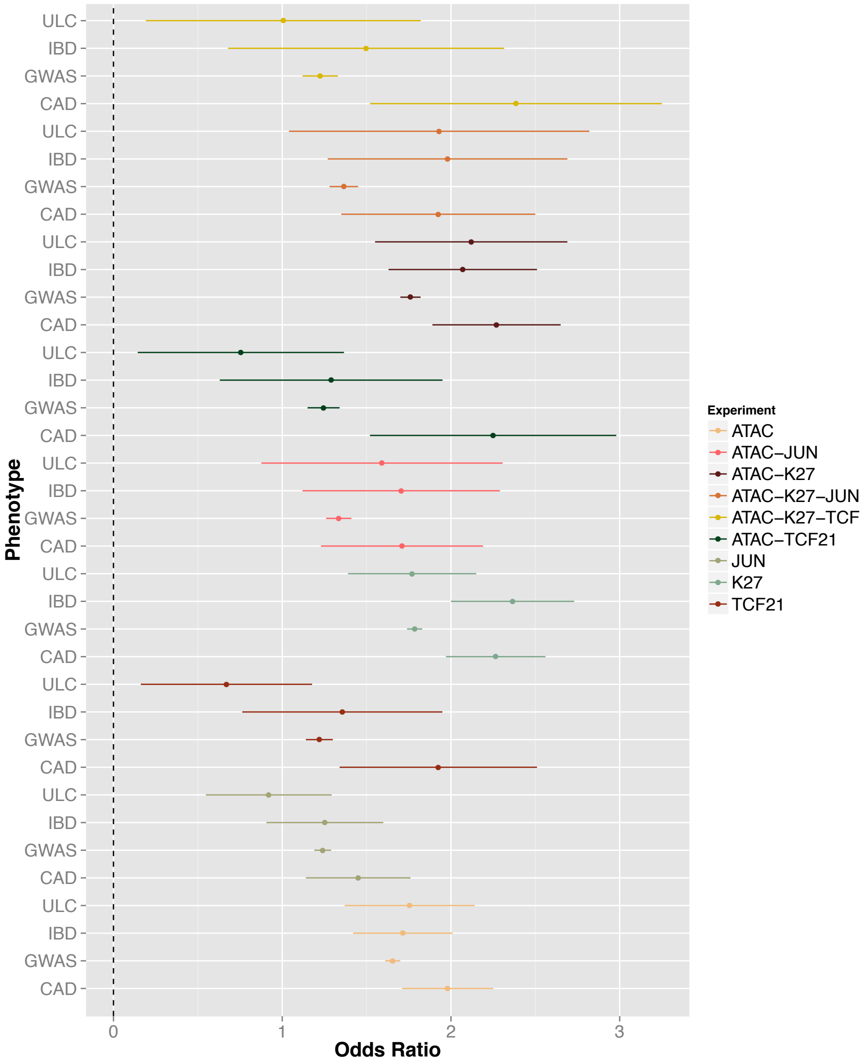

Supplementary Figure 5. Enrichment of CAD variants in individual and overlapping HCASMC datasets. Forest plot depicting odds ratio (OR) of significantly enriched loci (lead SNPs and LD SNPs) for coronary artery disease (CAD), inflammatory bowel disease (IBD), Ulcerative colitis (UC), or complete GWAS catalog within HCASMC regulatory regions (ATAC-seq, H3K27ac, TCF21, or JUN ChIP-seq), either individual or overlapping (color coded by overlap). Boxes represent mean OR and lateral lines represent 95% confidence intervals. Similar results observed in n=3 biological replicates.

Supplementary Fig. 6

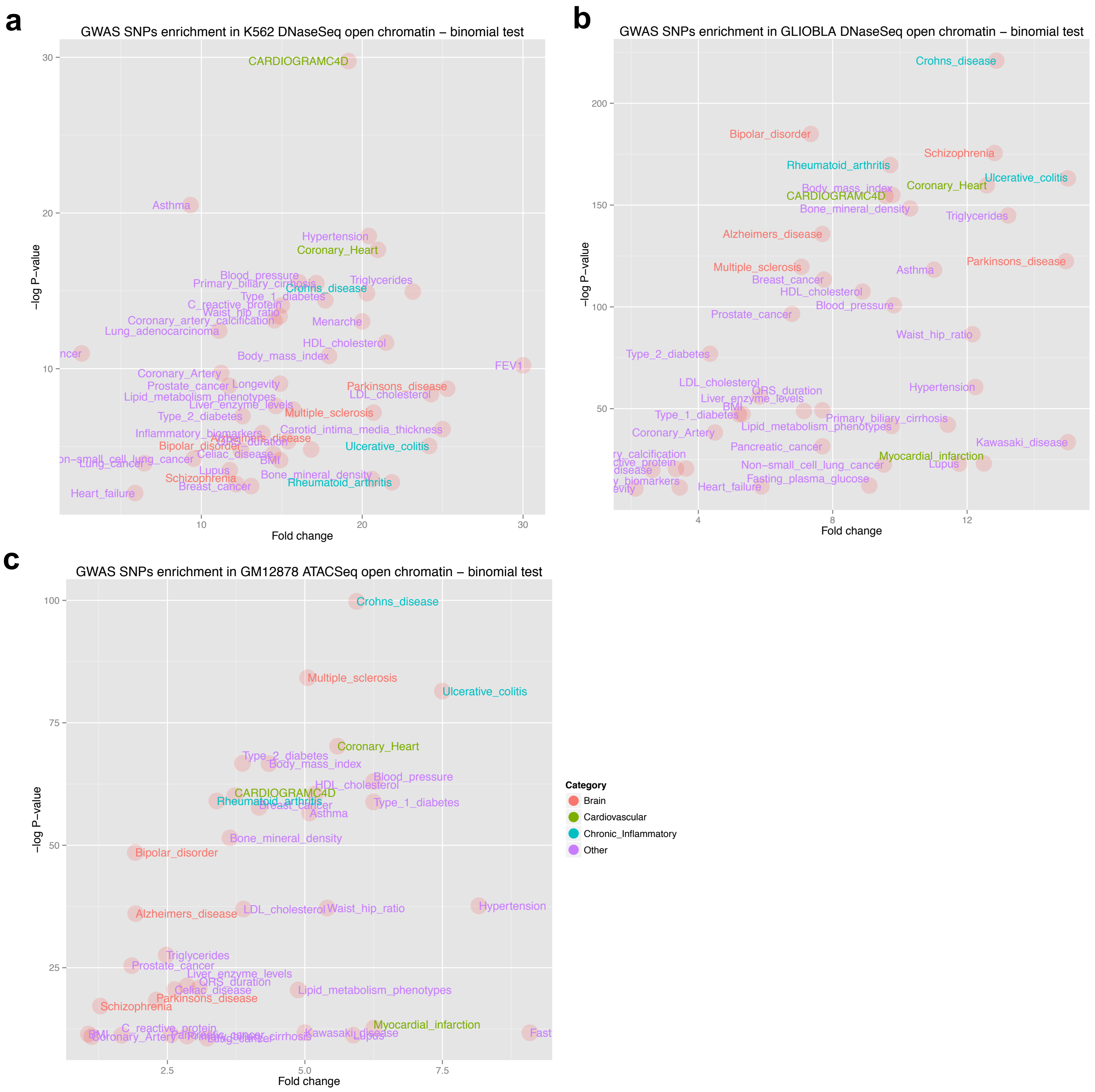

Supplementary Figure 6. GWAS SNP enrichment non-vascular open chromatin regions. (**a**) Two-dimensional scatter plot of GWAS SNP enrichment in K562 DNase-seq open chromatin regions using a binomial test, highlighting enrichment for brain disorders (e.g. Bipolar and Schizophrenia; Red), chronic inflammatory diseases (e.g. Rheumatoid arthritis, Crohns disease, Ulcerative colitis; Green), and CARDIOGRAMplusC4D (Purple) based coronary artery disease. (**b**) Similar plot showing enrichment in Glioblastoma DNase-seq open chromatin regions, showing increased enrichment of brain disorder and chronic inflammatory SNPs. (**c**) GM12878 lymphoblastoid ATAC-seq open chromatin regions, showing reduced enrichment of CARDIOGRAMplusC4D SNPs and brain disorders (Bipolar and Schizophrenia) suggesting both context-dependent regulatory effects and shared etiology between inflammatory disease phenotypes.

Supplementary Fig. 7

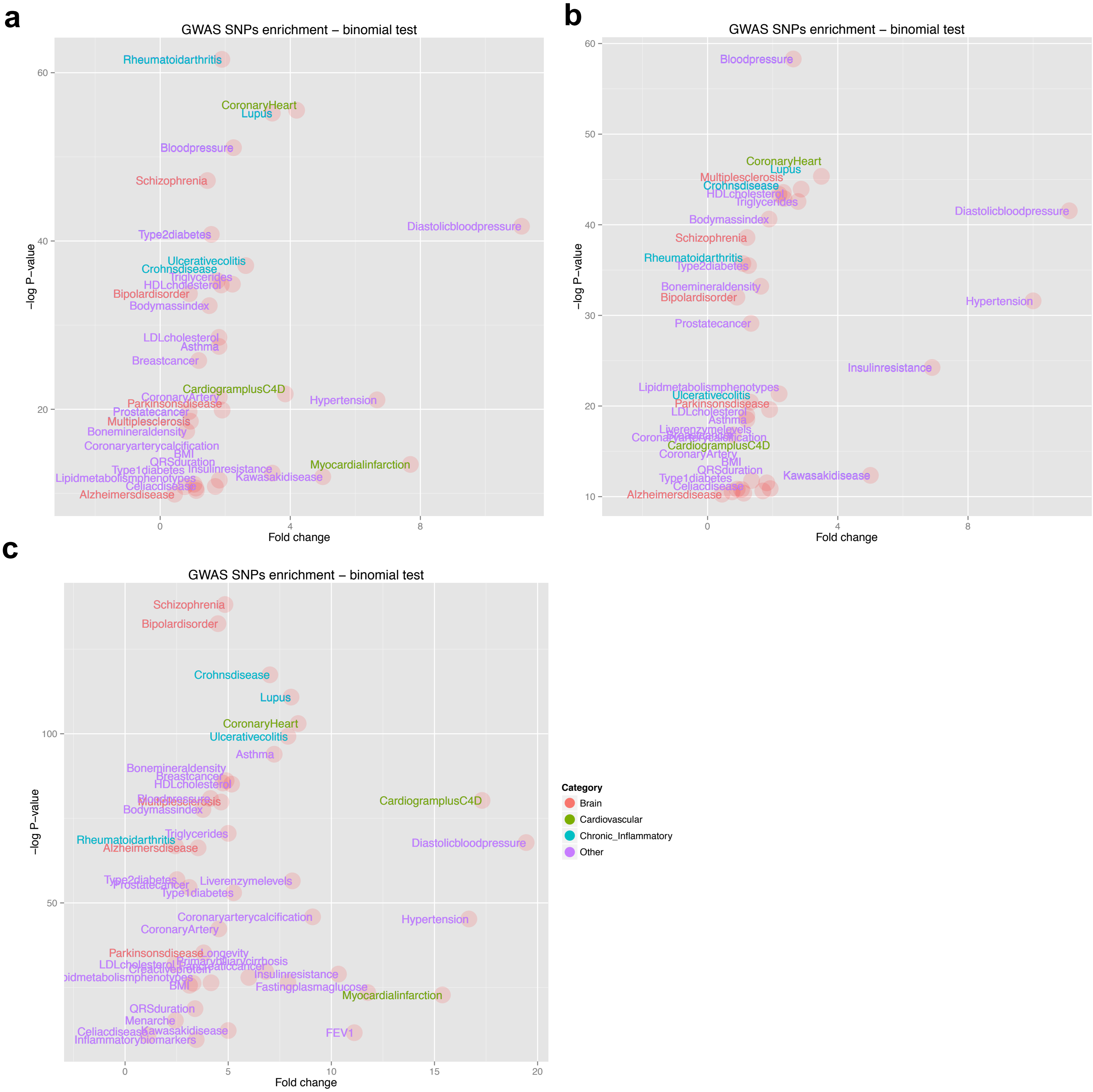

Supplementary Figure 7. **GWAS SNP enrichment in cardiovascular open chromatin regions and histone modification H3K27ac.** (**a**) Two-dimensional scatter plot of GWAS SNP enrichment in ATAC-Seq open chromatin regions from normal artery using a binomial test, highlighting enrichment for brain disorders (e.g. Bipolar and Schizophrenia; Red), chronic inflammatory diseases (e.g. Rheumatoid arthritis, Crohns disease, Ulcerative colitis, Lupus; Green), and CARDIOGRAMplusC4D (Purple) SNPs. (**b**) Similar plot showing enrichment in ATAC-Seq open chromatin regions from athero artery, showing increased enrichment of Coronary Heart Disease, Blood Pressure and Diastolic Blood Pressure SNPs. (**c**) HCASMC H3K27ac, showing increased enrichment of brain disorders (Bipolar and Schizophrenia), immune- disorders and Coronary Heart Disease suggesting shared etiology and pathways between these disease phenotypes.

Supplementary Fig. 8

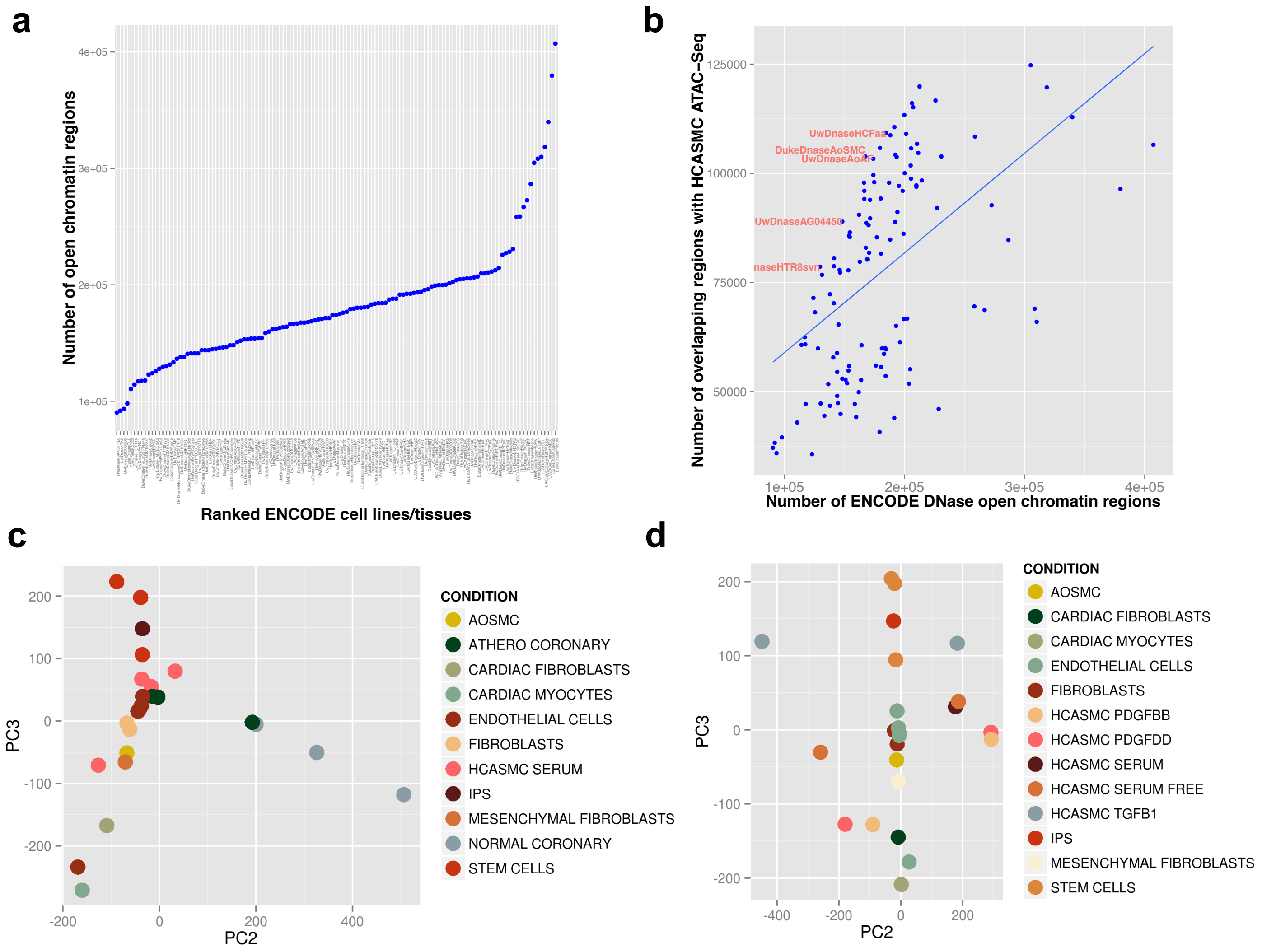

Supplementary Figure 8. Comparison of open chromatin regions in HCASMC and ENCODE data-sets. (**a**) Scatter plot of the total number of DNase hypersensitive open chromatin regions across the ENCODE catalog of cell types (n=125). Notably the majority of cell types contain between 100-200K open chromatin regions, while outliers with >200K regions represent less differentiated or immortalized cell types. (**b**) Scatter plot of overlapping ENCODE and HCASMC ATAC-seq open chromatin regions versus total ENCODE DNase regions. Two populations of cell types (separated by blue line) may reflect the heterogeneity of HCASMC during various stages of differentiation. We calculated the ratio of number of overlapped regions vs total open chromatin regions, and among those that have the 5 highest ratios are cardiac and cardiovascular tissues - aortic smooth muscle cells (AoSMC), aortic adventitial fibroblasts (AoAF) and cardiac fibroblasts HCF. (**c**) Principal component analysis (PCA) of normalized ENCODE DNase-seq open chromatin regions for select cell types and ATAC-seq open chromatin regions of serum treated HCASMC and normal and diseased human coronary artery tissues. Principal component 1 was excluded from the plot as it depicted batch effect between experiments. Principal component 2 separates normal and diseased coronary artery tissues. Principal component 3 separates the cells in the direction from undifferentiated stem cells and induced pluripotent stem cells (iPSC) to highly differentiated cardiac myocytes and brain derived endothelial cells.

Supplementary Fig. 9

a Normal coronary artery

	Motif	Rank	P-value	% Targets	% Bkgd
BATF	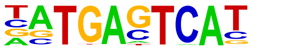	1	1e-1759	13.2	2.7
BORIS	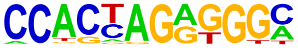	2	1e-1366	6.0	0.6
ATF1	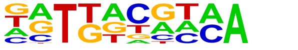	3	1e-410	21.8	13.5
ETV1	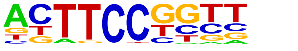	4	1e-346	21.5	13.8
NFIC	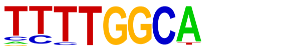	5	1e-332	27.3	18.9
MEF2C	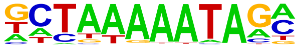	6	1e-280	3.5	1.1
TEAD4	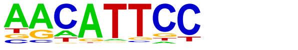	7	1e-214	18.7	13.0
KLF5	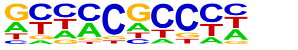	8	1e-173	13.1	8.7
SRF	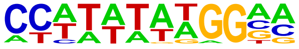	9	1e-162	3.3	1.4
NFY	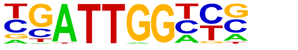	10	1e-154	6.3	3.5

b Athero coronary artery

	Motif	Rank	P-value	% Targets	% Bkgd
FRA1	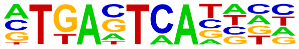	1	1e-991	12.7	3.6
BORIS	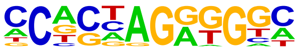	2	1e-688	4.6	0.7
NFIC	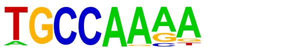	3	1e-304	28.8	19.9
MEF2C	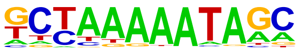	4	1e-298	7.1	2.9
TEAD2	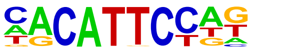	5	1e-253	9.8	5.0
ELK4	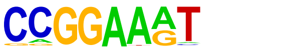	6	1e-232	23.0	15.8
ATF1	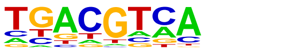	7	1e-204	12.8	7.7
KLF5	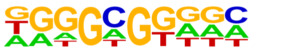	8	1e-158	22.4	16.5
SRF	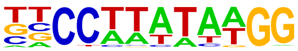	9	1e-105	1.3	0.4
NFY	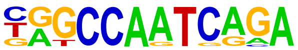	10	1e-100	4.9	2.7

Supplementary Figure 9. De novo motif enrichment analysis of open chromatin regions in normal and atherosclerotic human coronary arteries. (**a**) Top 10 enriched transcription factor binding motifs in normal ex vivo coronary artery derived ATAC-seq open chromatin regions. (**b**) Similar results using atherosclerotic coronary artery ATAC-seq open chromatin regions. P-values determined from cumulative binomial distribution using randomly assigned and GC content matched sequences as background regions. Results are representative of n=3 biological replicates per condition.

Supplementary Fig. 10

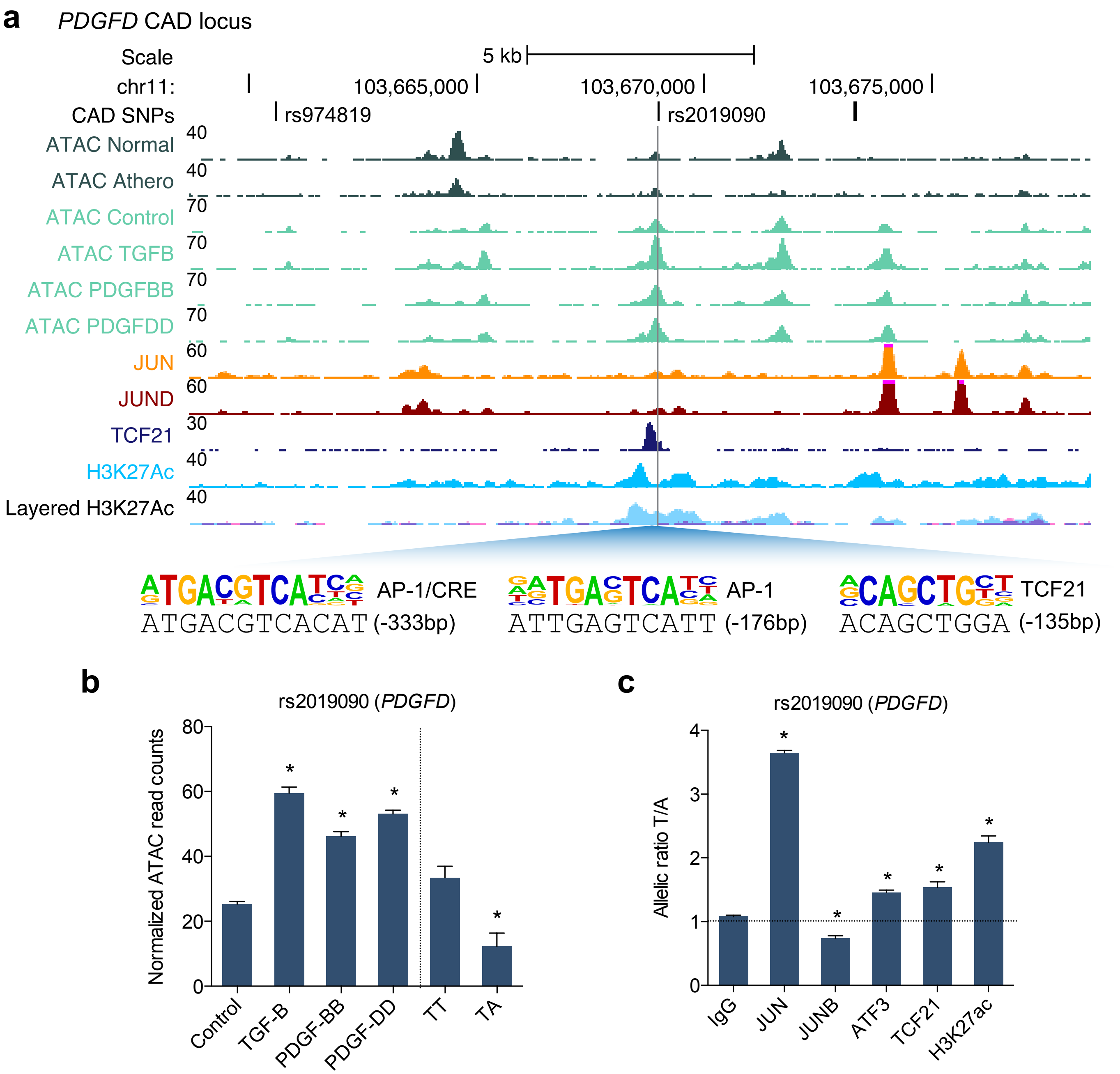

Supplementary Figure 10. AP-1 and TCF21 mediated regulatory mechanism at PDGFD CAD locus. (**a**) UCSC Browser screenshot of the PDGFD CAD locus at chromosome 11q22.3 highlighting the candidate causal variant, rs2019090, overlapping ATAC-seq open chromatin tracks in coronary tissue (n=3 biological replicates per condition) and HCASMC treated under various conditions (n=2 biological replicates per condition), TF binding ChIP-seq tracks for TCF21, JUN, and JUND, and active enhancer histone modification H3K27ac ChIP-seq (n=4 biological replicates), as well as ENCODE layered H3K27ac tracks for HUVEC (blue) and NHLF (purple) cells. Inset, consensus motifs are shown for AP-1, AP-1/CRE, and TCF21 with alignment to hg19 reference sequence and position relative to SNP rs2019090. (**b**) Normalized ATAC-seq read counts for HCASMC treated under various conditions and by genotype at rs2019090. Values represent mean ± SEM (n=2 biological replicates for stimulations and n=4 biological replicates for different genotypes). (**c**) Allele-specific ChIP (haploChIP) for AP-1 proteins (JUN, JUNB, ATF3), TCF21, and H3K27ac in HCASMC heterozygous at rs2019090. Values represent mean ± SEM of triplicates from a representative experiment (n=3 biological replicates). *P<0.01 versus Control, IgG or between two genotypes using an unpaired two-tailed t test with Welch's correction for unequal variances.

Supplementary Fig. 11

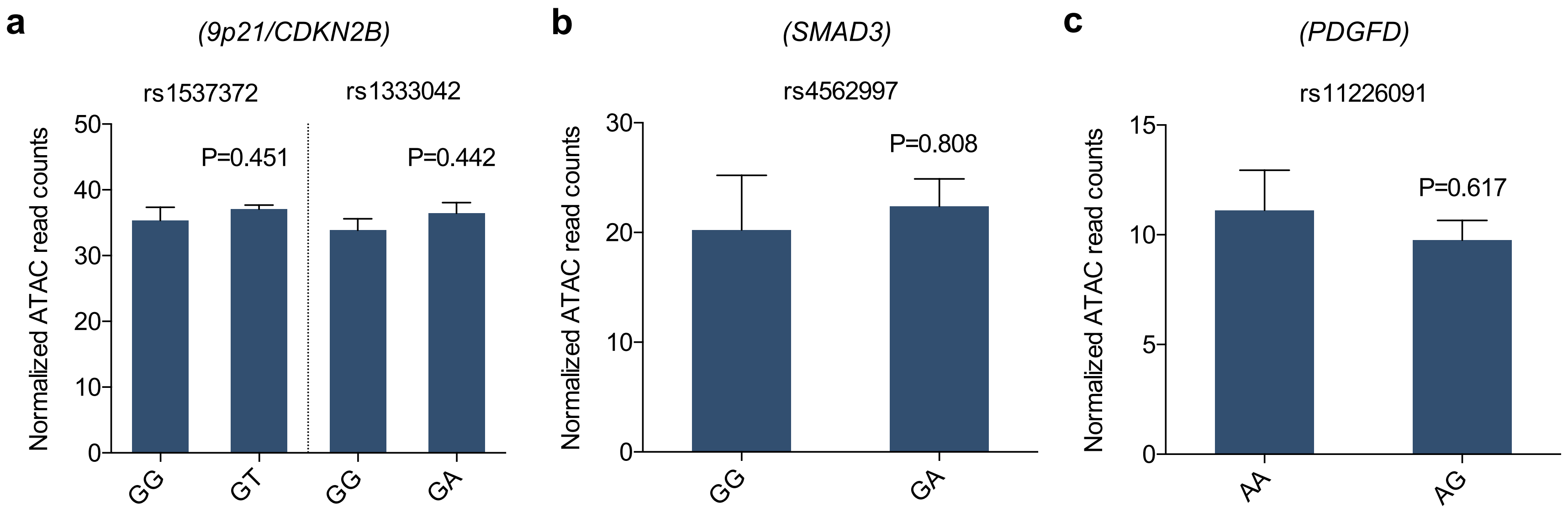

Supplementary Figure 11. Effect of LD SNPs on HCASMC chromatin accessibility. (**a**) Summary of normalized ATAC-seq read counts overlapping two SNPs, rs1537372 and rs1333042, in perfect LD (r2=1) with candidate causal SNP rs1537373 at 9p21/CDKN2B locus. (**b**) Normalized ATAC-seq read counts overlapping SNP rs4562997, in LD (r2=0.65) with candidate causal SNP rs17293632 at SMAD3 locus. (**c**) Normalized ATAC-seq read counts overlapping SNP rs11226091 in LD (r2=0.85) with candidate causal SNP rs2019090 at the PDGFD locus. Values represent mean ± SD (n = 3 biological replicates per genotype). P-values determined by unpaired two-tailed t test with Welch's correction for unequal variances.

Supplementary Fig. 12

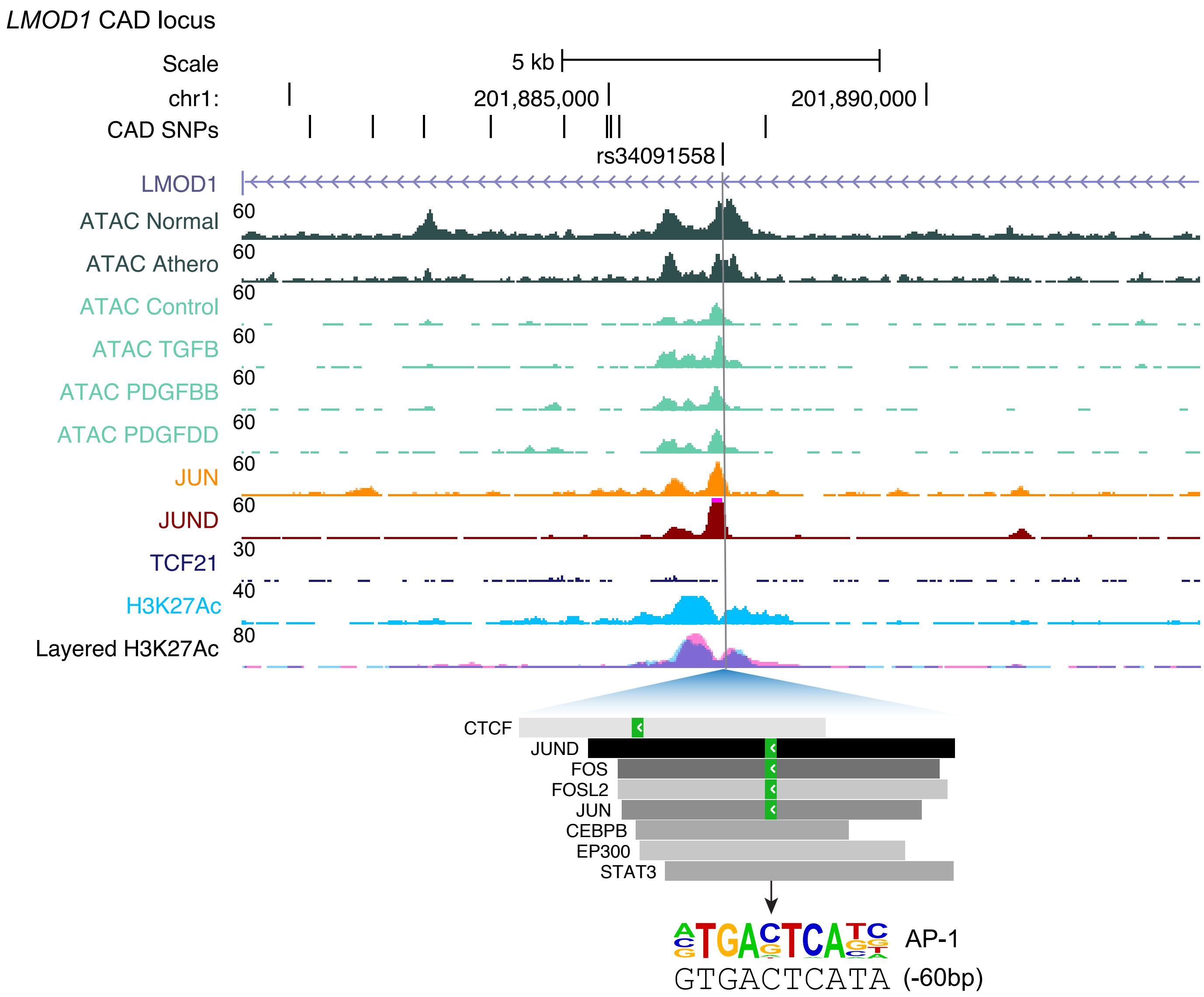

Supplementary Figure 12. AP-1 mediated regulatory mechanism at LMOD1 CAD locus. UCSC Browser screenshot of the LMOD1 CAD locus at chromosome 1q32.1 highlighting the candidate causal variant, rs34091558, overlapping ATAC-seq open chromatin tracks in coronary tissue ex vivo (n=3 biological replicates per condition) and HCASMC treated under various conditions (n=2 biological replicates per condition), TF binding ChIP-seq tracks for TCF21, JUN, and JUND, and active enhancer histone modification H3K27ac ChIP-seq (n=4 biological replicates), as well as ENCODE layered H3K-27ac tracks for HUVEC (blue) and NHLF (purple) cells. Genomic coordinates refer to hg19 assembly.

Supplementary Fig. 13

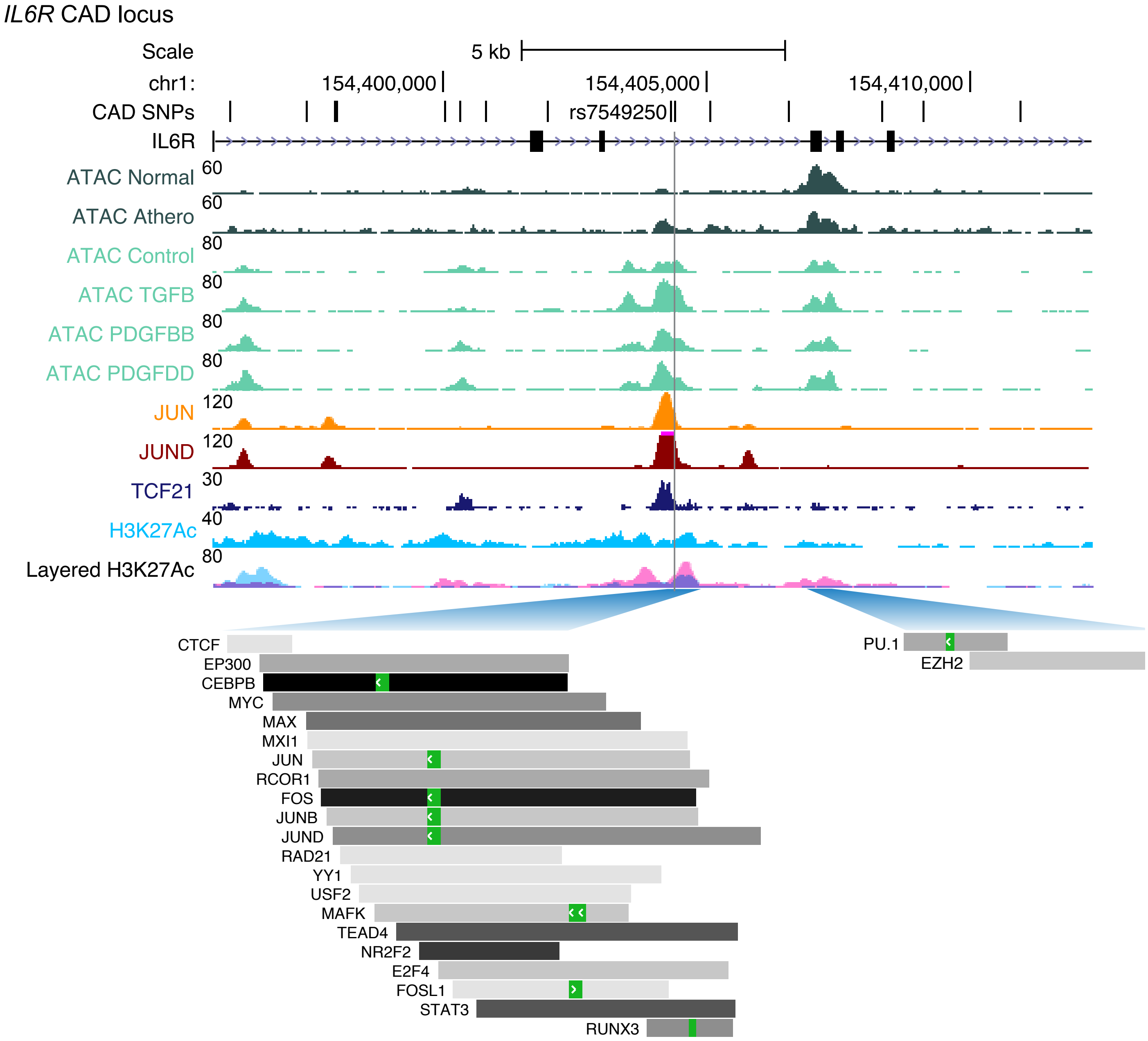

Supplementary Figure 13. AP-1 mediated regulatory mechanism at IL6R CAD locus.
UCSC Browser screenshot of the IL6R CAD locus at chromosome 1q21.3 highlighting the candidate causal variant, rs7549250, overlapping ATAC-seq open chromatin tracks in coronary tissue ex vivo (n=3 biological replicates per condition) and HCASMC treated under various conditions (n=2 biological replicates per conidition), TF binding ChIP-seq tracks for TCF21, JUN, and JUND, and active enhancer histone modification H3K27ac ChIP-seq (n=4 biological replicates), as well as ENCODE layered H3K-27ac tracks for HUVEC (blue) and NHLF (purple) cells. Inset, ENCODE ChIP-seq tracks shown for 0.5kb region surrounding rs7549250 as well as nearby open chromatin region which may reflect binding of pioneering and repressive TFs. Genomic coordinates refer to hg19 assembly.

Supplementary Fig. 14

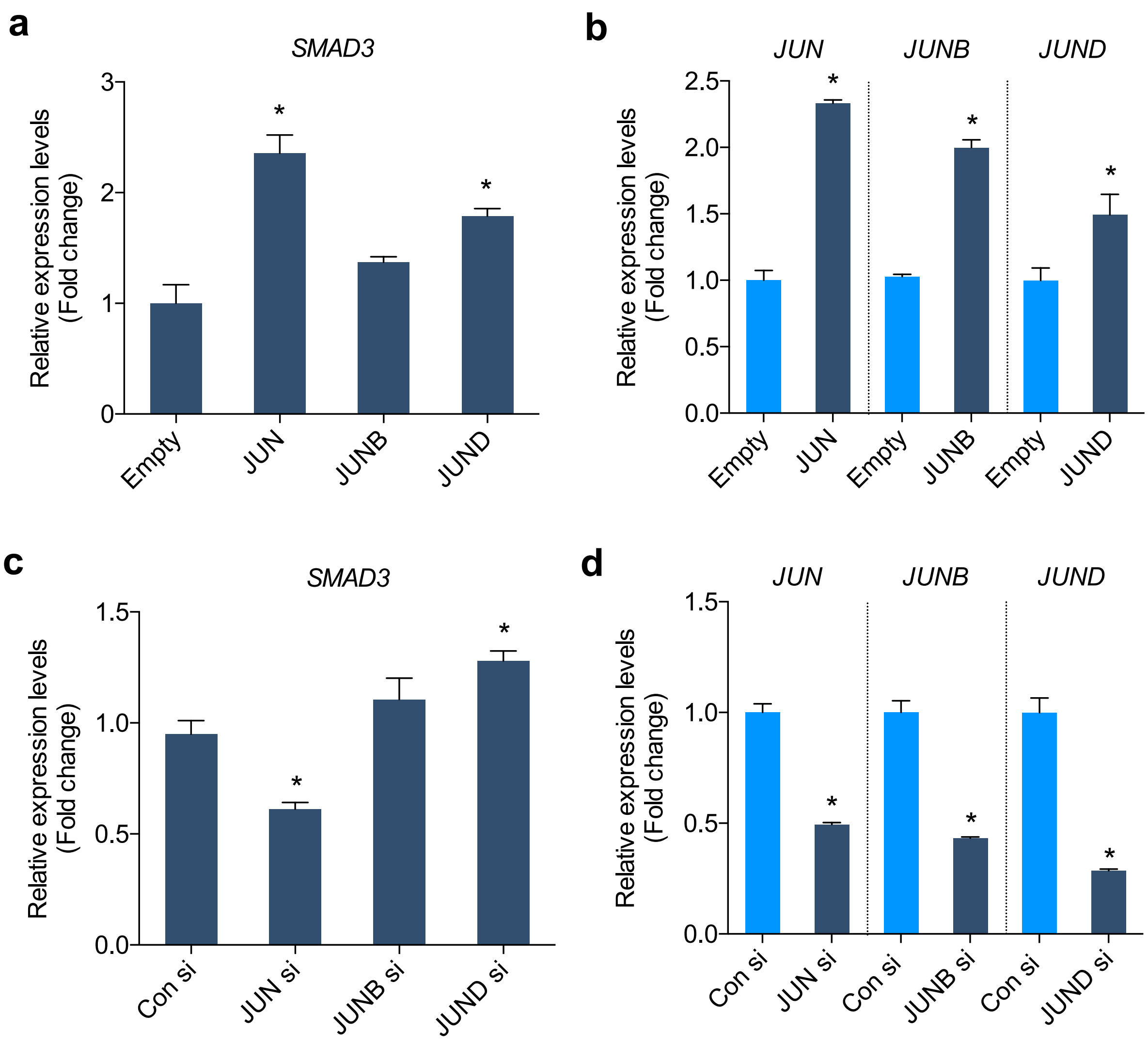

Supplementary Figure 14. Effects of AP-1 overexpression and silencing on endogenous AP-1 and SMAD3 levels in HCASMC. TaqMan quantitative PCR (qPCR) results of (**a**) human SMAD3 or (**b**) AP-1 (JUN, JUNB, or JUND) expression levels in AP-1 plasmid transfected HCASMC after 24h. (**c**) SMAD3 or (d) AP-1 expression levels in AP-1 siRNA transfected HCASMC after 48h. Values represent mean ± SEM of triplicates from a representative experiment (n=3 biological replicates). *P<0.01 versus Empty vector control or Con si treatment.

Supplementary Fig. 15

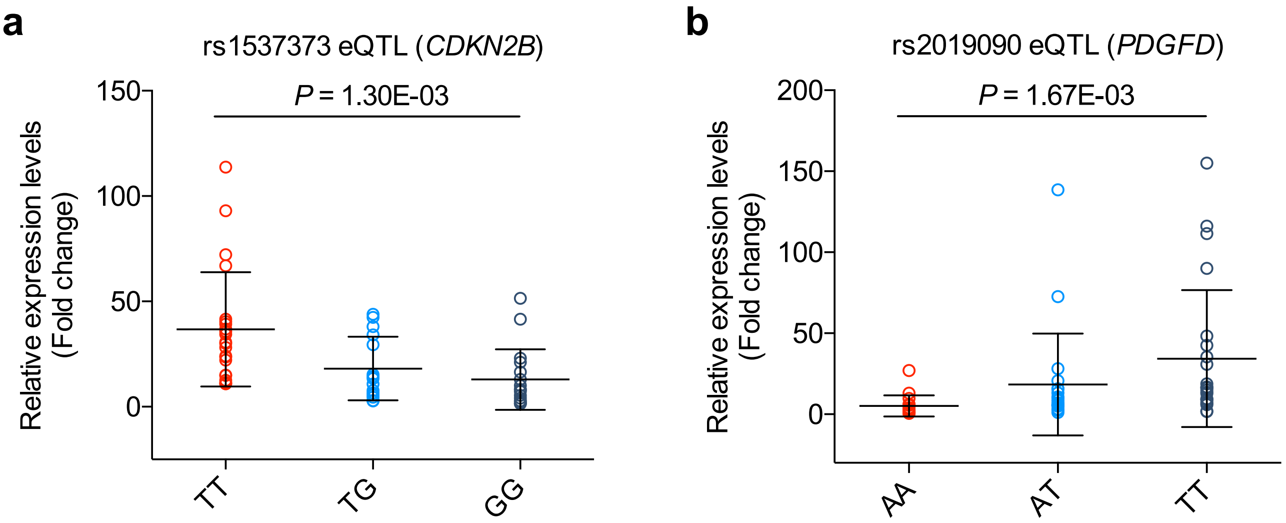

Supplementary Figure 15. Expression quantitative trait loci validation in HCASMC for CDKN2B and PDGFD candidate variants. (**a**) CDKN2B gene expression levels in HCASMC with respect to genotype at rs1537373, expressed as ΔΔCt values normalized to GAPDH levels (fold change). (**b**) PDGFD gene expression levels in HCASMC with respect to genotype at rs2019090, expressed as ΔΔCt values normalized to GAPDH levels (fold change). Values represent mean ± SEM of triplicates (n=64 independent donors/biological replicates). P-values calculated using a Welch's unequal variances t test.

Supplementary Fig. 16

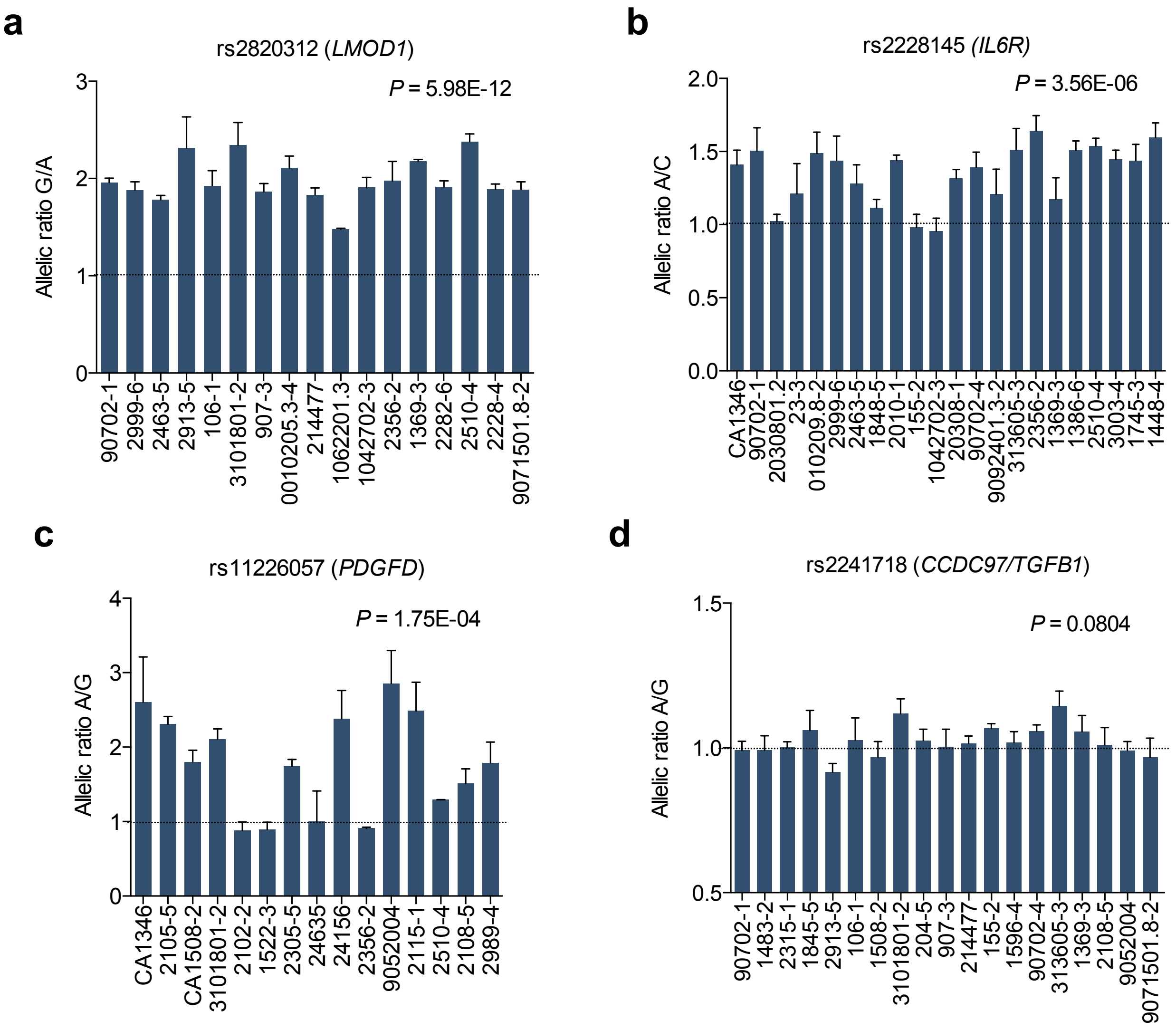

Supplementary Figure 16. Allelic expression imbalance in HCASMC for candidate CAD regulatory variants. (**a-d**) Allelic expression imbalance (AEI) for four candidate CAD regulatory SNPs detected by TaqMan qPCR of coding or UTR SNPs as a proxy (D'=0.8-1.0). Values represent mean ± SEM of triplicates for relative cDNA allelic ratio in individual donor HCASMC, determined to be heterozygous for each SNP. P-values shown represent comparison of AEI from all samples versus expected allelic ratio of 1.0 using a Welch's unequal variances t test.

Supplementary Fig. 17

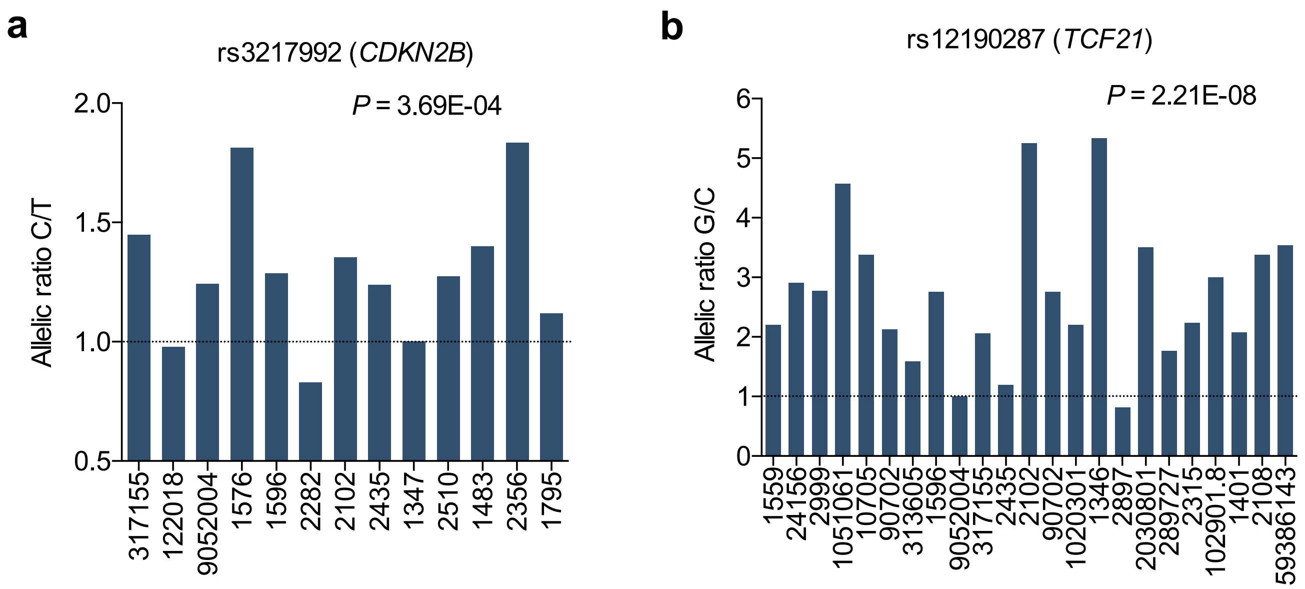

Supplementary Figure 17. Allelic expression imbalance in HCASMC for candidate CAD regulatory variants using RNA-seq. (**a**) Allelic imbalance for candidate variant at CDKN2B detected by RNA-seq reads in HCASMC from individuals heterozygous at 3'-UTR SNP rs3217992 (D'=0.75) as a proxy. (**b**) Allelic imbalance for lead SNP at TCF21 detected by RNA-seq reads in HCASMC from individuals heterozygous at rs12190287 (located in 3'-UTR of longer variant 1 of TCF21). Allelic ratios determined from ~30X depth sequencing reads using >10 reads covering heterozygous sites. P-values shown represent comparison of AEI from all samples versus expected allelic ratio of 1.0 using a Welch's unequal variances t test.

Supplementary Fig. 18

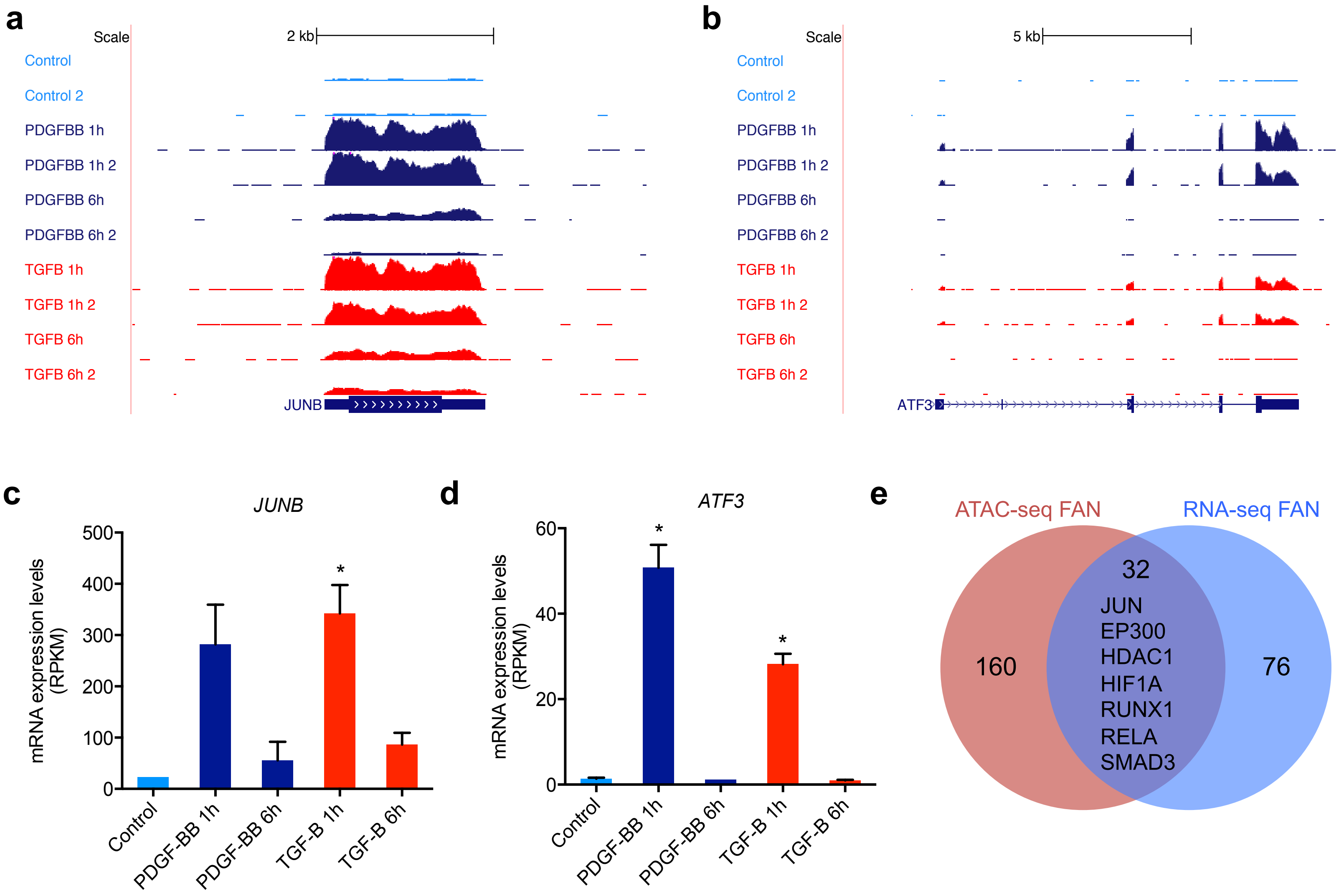

Supplementary Figure 18. Differential expression of immediate early response genes in stimulated HCASMC. (**a**) UCSC screenshot showing early induction (1hr) of AP-1 gene JUNB in TGF-β and PDGF-BB stimulated HCASMC via RNA-seq. Modest induction was observed at later timepoints (6hr) (**b**) UCSC screenshot showing similar induction of AP-1 gene ATF3 in stimulated HCASMC. (**c, d**) Quantitation of RNA-seq counts shown as RPKM values (n=2 independent donors/biological replicates). P-values represent mean ± SEM versus Control. (**e**) Venn diagram showing the overlap of differentially expressed genes and those near open chromatin regions in PDGF-BB stimulated HCASMC that were both annotated using functional association networks (FANs). Relevant TFs are listed among the 32 overlapping genes.

Supplementary Fig. 19

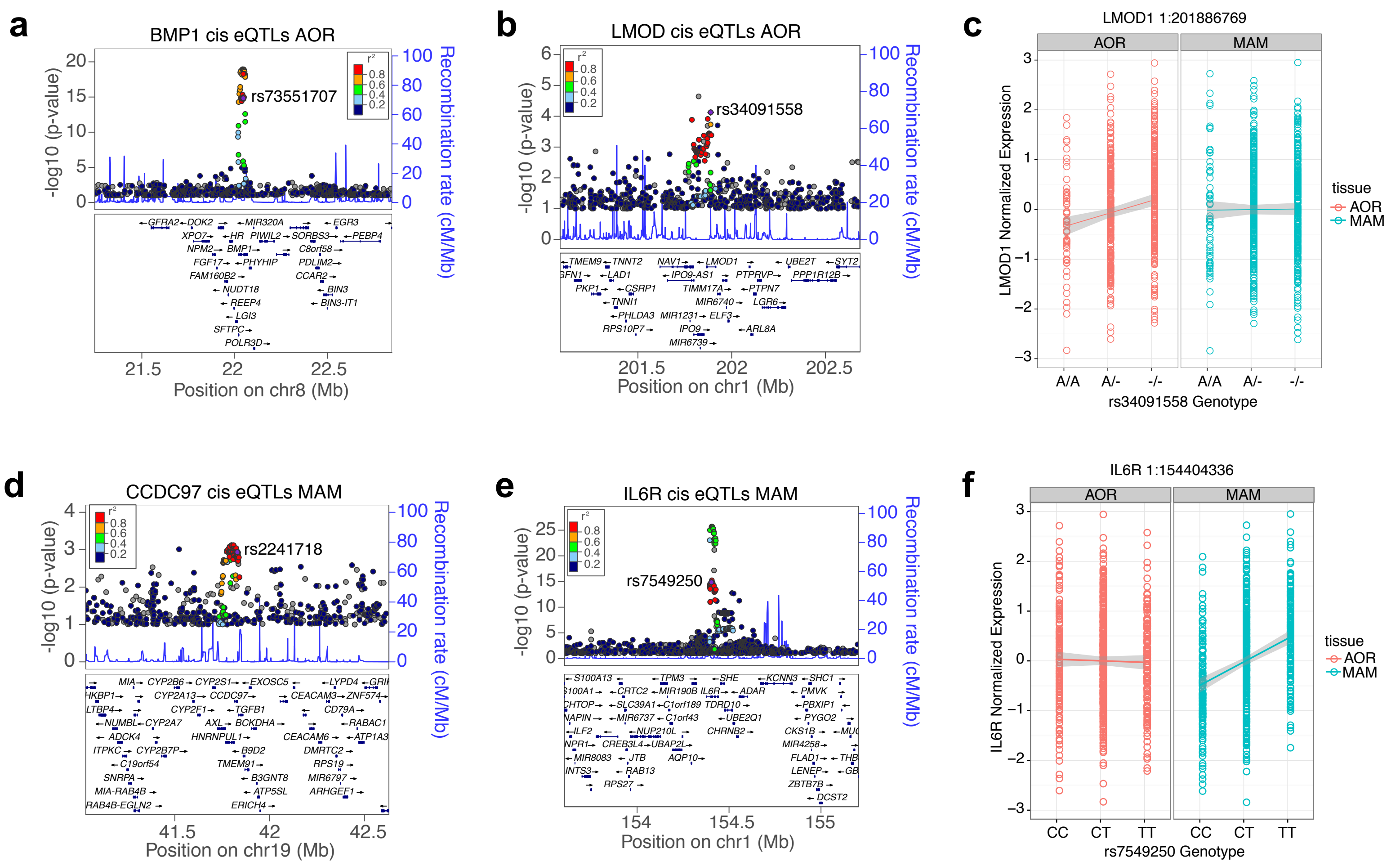

Supplementary Figure 19. Validation of LMOD1 and IL6R candidate variants in external CAD eQTL datasets. (**a-b**) Distribution of all cis-eQTLs called for LMOD1 in diseased human aorta (n=513 independent donors/biological replicates) and mammary artery (n=528 independent donors/biological replicates) tissues, respectively. The dashed line represents the gene of interest, and the blue and red dots represent the max SNP (most significant eQTL associated with LMOD1) and the SNP of interest, respectively. (**b**) For mammary artery tissue, the SNP of interest was not identified as an eQTL for LMOD1. (**c**) For each genotype the normalized expression of LMOD1 is shown as well as the linear regression line. (**d-e**) Distribution of all cis eQTLs called for IL6R in aorta and mammary artery tissues, respectively. The dashed line represents the gene of interest, with the blue and red dots being the max SNP (most significant eQTL associated with IL6R) and the SNP of interest, respectively. (**d**) For aortic tissue, the SNP of interest was not identified as an eQTL for IL6R. (**f**) For each genotype the normalized expression of IL6R is shown as well as the linear regression line. Log10(p-values) and log2 normalized expression levels determined as described in Methods. AOR: aorta; MAM: mammary artery. Genomic coordinates refer to hg19 assembly.

Supplementary Fig. 20

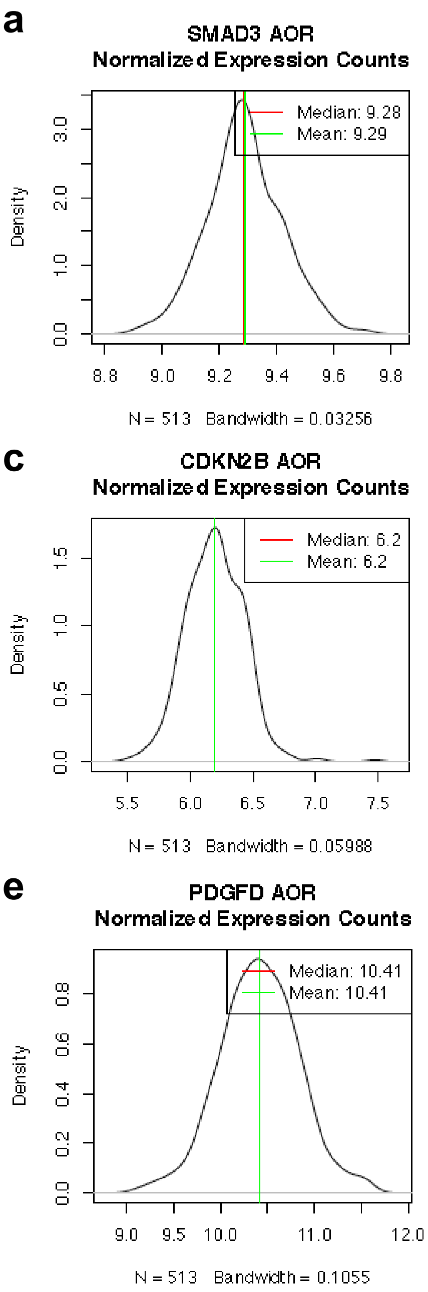

Supplementary Figure 20. Normalized RNA-seq expression read counts at select candidate CAD loci. (**a, c, e**) Normalized read counts for SMAD3, CDKN2B, and PDGFD genes in atherosclerotic aortic tissue (AOR) from STARNET cohort. (**b, d, f**) Normalized read counts for SMAD3, CDKN2B, and PDGFD genes in mammary artery tissue (MAM) from STARNET cohort. Normalization of read counts was performed using the EDAseq R/Bioconductor package as described in the Methods.
